# Influence of sex, cigarette smoking and airway inflammation on treatable traits in CBIOPRED severe asthma

**DOI:** 10.1002/clt2.12189

**Published:** 2022-09-15

**Authors:** Cong Dong, Xiaojing Yang, Wei Luo, Ethan Fan, Nkouibert Pryseley Assam, Jian Kang, Yunhui Zhang, Mao Huang, Jinfu Xu, Kewu Huang, Qiang Li, Xiangyan Zhang, Jianping Zhao, Xiaoxia Liu, Shenghua Sun, Huaping Tang, Bei He, Shaoxi Cai, Ping Chen, Chunhua Wei, Guangfa Wang, Ping Chen, Lixin Xie, Jiangtao Lin, Yuling Tang, Zhihai Han, Xiuhua Fu, Changzheng Wang, Huahao Shen, Meiling Jin, Lei Zhu, Guochao Shi, Zhongmin Qiu, Zhongguang Wen, Wei Gu, Kian Fan Chung, Qingling Zhang, Nanshan Zhong, C‒BIOPRED consortium

**Affiliations:** ^1^ Pulmonary and Critical Care Medicine Guangzhou Institute of Respiratory Health National Clinical Research Center for Respiratory Disease National Center for Respiratory Medicine State Key Laboratory of Respiratory Diseases The First Affiliated Hospital of Guangzhou Medical University Guangzhou China; ^2^ AstraZeneca Shanghai China; ^3^ Department of Respiratory Medicine The First Hospital of China Medical University Shenyang China; ^4^ Department of Respiratory Medicine The First People's Hospital of Yunnan Province Kunming China; ^5^ Department of Respiratory Medicine Jiangsu Province Hospital Nanjing China; ^6^ Department of Respiratory Medicine Shanghai Pulmonary Hospital Shanghai China; ^7^ Department of Respiratory Medicine Beijing Chao‐Yang Hospital Beijing China; ^8^ Department of Respiratory Medicine Shanghai General Hospital Shanghai China; ^9^ Department of Respiratory Medicine Guizhou Province People's Hospital Guiyang China; ^10^ Department of Respiratory Medicine Tongji Hospital Tongji Medical College Huazhong University of Science and Technology Wuhan China; ^11^ Department of Respiratory Medicine Beijing Friendship Hospital Capital Medical University Beijing China; ^12^ Department of Respiratory Medicine The Third Xiangya Hospital of Central South University Changsha China; ^13^ Department of Respiratory Medicine Qingdao Municipal Hospital Qingdao China; ^14^ Department of Respiratory Medicine Peking University Third Hospital Beijing China; ^15^ Department of Respiratory Medicine Nanfang Hospital of Southern Medical University Guangzhou China; ^16^ Department of Respiratory Medicine The General Hospital of Shenyang Military Shenyang China; ^17^ Department of Respiratory Medicine Weifang Asthma Hospital Weifang China; ^18^ Department of Respiratory Medicine Peking University First Hospital Beijing China; ^19^ Department of Respiratory Medicine The Second Xiangya Hospital of Central South University Changsha China; ^20^ College of Pulmonary and Critical Care Medicine Chinese PLA General Hospital Beijing China; ^21^ Department of Respiratory Medicine China‐Japan Friendship Hospital Beijing China; ^22^ Department of Respiratory Medicine The First Hospital of Changsha Changsha China; ^23^ Department of Respiratory Medicine Navy General Hospital Beijing China; ^24^ Department of Respiratory Medicine The Affiliated Hospital of Inner Mongolia Medical University Huhhot China; ^25^ Department of Respiratory Medicine Xinqiao Hospital Army Military Medical University Chongqing China; ^26^ Department of Respiratory Medicine The Second Affiliated Hospital of Zhejiang University School of Medicine Hangzhou China; ^27^ Department of Respiratory Medicine Zhongshan Hospital Shanghai China; ^28^ Department of Respiratory Medicine Ruijin Hospital Shanghai Jiaotong University School of Medicine Shanghai China; ^29^ Department of Respiratory Medicine Shanghai Tongji Hospital Shanghai China; ^30^ Department of Respiratory Medicine The First Affiliated Hospital of PLA General Hospital Beijing China; ^31^ Department of Respiratory Nanjing First Hospital Nanjing Medical University Nanjing China; ^32^ Experimental Studies National Heart and Lung Institute Imperial College London, & Royal Brompton & Harefield NHS Foundation Trust London UK

**Keywords:** baseline and longitudinal, gender, severe asthma, smoking

To the Editor

Asthma presents a major public health challenge in China, where it affects 45.7 million adults with an estimated prevalence of 4.2%; 26.2% of adults with asthma have airflow limitation.^1^ Severe asthma is recognised in China with the recent publication of an expert consensus guidelines for the diagnosis and management of severe asthma.^2^ Severe asthma has been defined as asthma that requires treatment with high dose inhaled corticosteroids plus a second controller and/or systemic corticosteroids to prevent it from becoming “uncontrolled” or that remains “uncontrolled” despite this therapy.^3^ More recently, the identification of a higher number of treatable traits in severe asthma compared to mild‐moderate asthma has facilitated a strategy for the management of airways disease.^4^ However, the influence of various factors on the major treatable traits such as airflow obstruction and exacerbations is unclear. Therefore, we examined the role of sex and cigarette smoking on these traits and also their link to airway inflammation measured by inflammatory cell counts in induced sputum samples.

In the Chinese Biomarkers for the Prediction of Respiratory Disease Outcomes (C‐BIOPRED) cohort of severe asthma, we compared the parameters of male to female non‐smoking severe asthma (NSA) and male current smoking severe asthma (CSA) and ex‐smoking (ESA) severe asthma at entry and at 1 year (Figure 1). Participants attended severe asthma university clinics where the diagnosis was ascertained and asthma treatment with adherence optimised. Non‐smoking severe asthma were non‐smokers for the past 12 months, with <5 pack‐year smoking. CSA and ESA, exclusively male, had a smoking history of >5 pack‐years. Severe asthma was defined according to European Respiratory Society and American Thoracic Society guidelines.^3^

**FIGURE 1:**
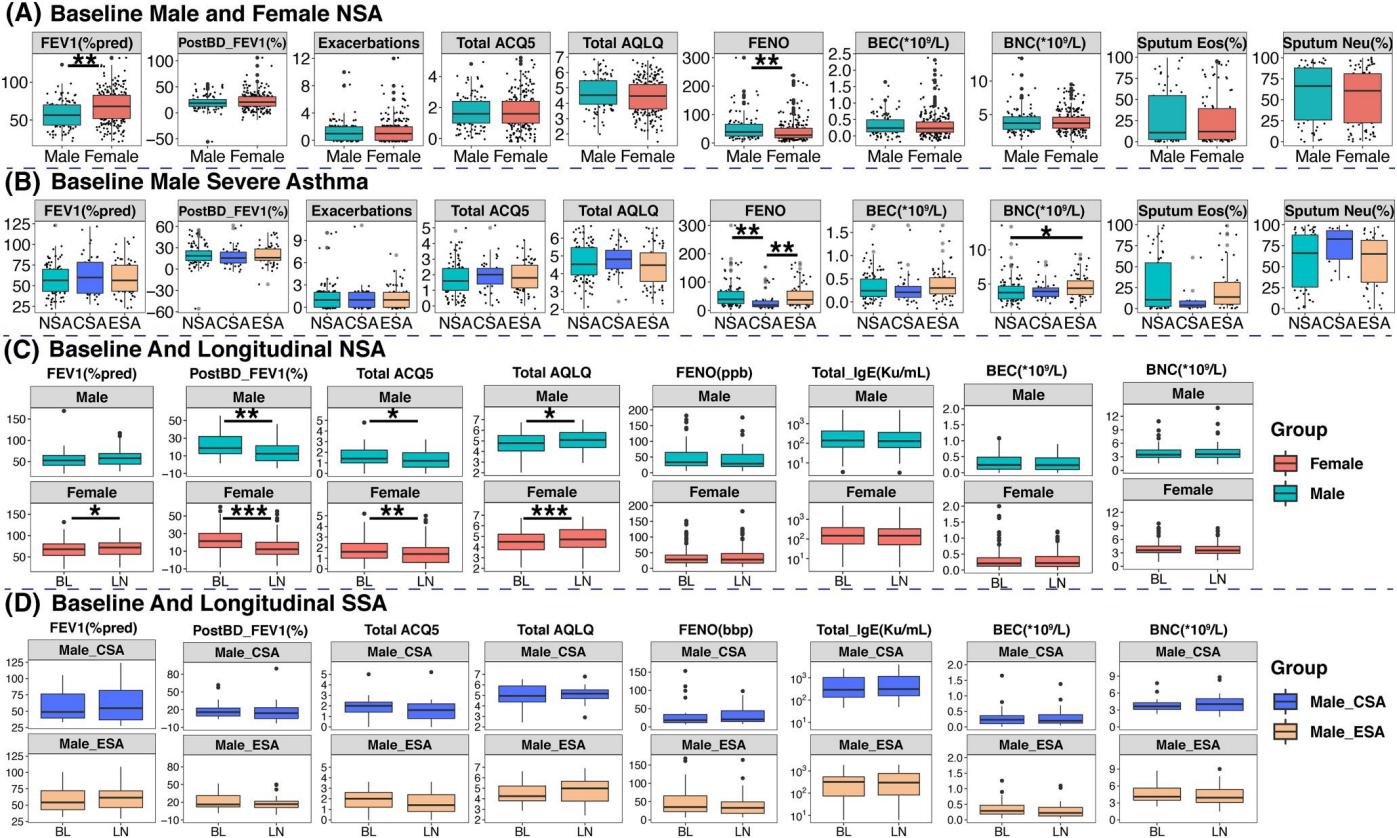
Panel A: Median (25%–75% Interquartile range [IQR]) values comparing male and female non‐smoking severe asthma (NSA). Panel B: Median (25%–75% IQR) values of male non‐smoking severe asthma (NSA), male current smoking severe asthma (CSA) and ex‐smoking (ESA) severe asthma at baseline. Panel C: Median (25%–75% IQR) values of male and female non‐smoking severe asthma (NSA) at baseline (BL) and at longitudinal (LN) follow‐up at 1 year. Panel D: Median (25%–75% IQR) values of male smoking severe asthma (SSA) including male CSA, ESA severe asthma at baseline (BL) and at LN follow‐up at 1 year. ACQ5, Asthma Control questionnaire five questions score; AQLQ, Asthma quality of life questionnaire score; BEC, Blood eosinophil count; BNC, Blood neutrophil count; Eos, eosinophil; FeNO, Fractional exhaled nitric oxide; FEV_1_, Forced expiratory volume in one second; IgE, Immunoglobulin E; Neu, neutrophil; ppb, parts per billion; PostBD‐FEV_1_ (%), percent improvement in FEV_1_ after salbutamol bronchodilator

There were no differences in clinical parameters and asthma control between NSA male and female but the pre‐bronchodilator FEV_1_ (% predicted) were lower in male compared to female participants while the bronchodilator response was similar in both groups. Fractional exhaled nitric oxide (FeNO) levels were higher in male (39.0 ppb) compared to 28.0 ppb in female (*p* = 0.001), but without any differences in sputum or blood eosinophil counts (Figure 1A,B; Supplementary Table S1).

Male NSA group was younger than CSA and ESA groups with the diagnosis of asthma made at a younger age (Supplementary Table S1). There was no difference in the number of exacerbations but greater healthcare resource utilization in ESA and NSA (18.5% and 21.8%, respectively) compared to 4.9% in CSA. Atopy incidence was highest in CSA (70%) compared to ESA and NSA (46.4% and 45.9%, respectively). There was no difference in the Asthma Control Questionnaire (ACQ) and total questionnaire of asthma quality of life in adults (AQLQ) scores apart from activity limitation score being lowest in ESA (Supplementary Table S2). There were no differences in baseline spirometric measurements and post‐bronchodilator FEV_1_ was similar in all groups. There was a lower FeNO in CSA (18.50 ppb) compared to ESA and NSA (37.00 and 39.00 ppb, respectively) (Supplementary Table S1), with no difference in blood eosinophil counts, but neutrophil counts were highest in ESA. CSA showed a trend towards higher sputum neutrophil (%) and lower eosinophil (%) counts compared to ESA and CSA (*p* = 0.08 and 0.07, respectively).

We examined the potential influence of inflammatory markers on airflow obstruction and exacerbations by performing Spearman's correlation coefficients analysis (Figure 2). FEV_1_ (% predicted) and FEV1/Forced Vital Capacity ratio were negatively correlated with sputum neutrophil (%) in male and female NSA, but not in CSA and ESA (Figure 2). Exacerbations were negatively correlated with sputum neutrophils (%) in female NSA and positively correlated with sputum eosinophils in male and female NSA. Fractional exhaled nitric oxide was positively correlated with sputum eosinophils in male and female NSA, and with the improvement in post‐bronchodilator FEV1 (%) in CSA (*r* = 0.926; *p* < 0.001).

**FIGURE 2:**
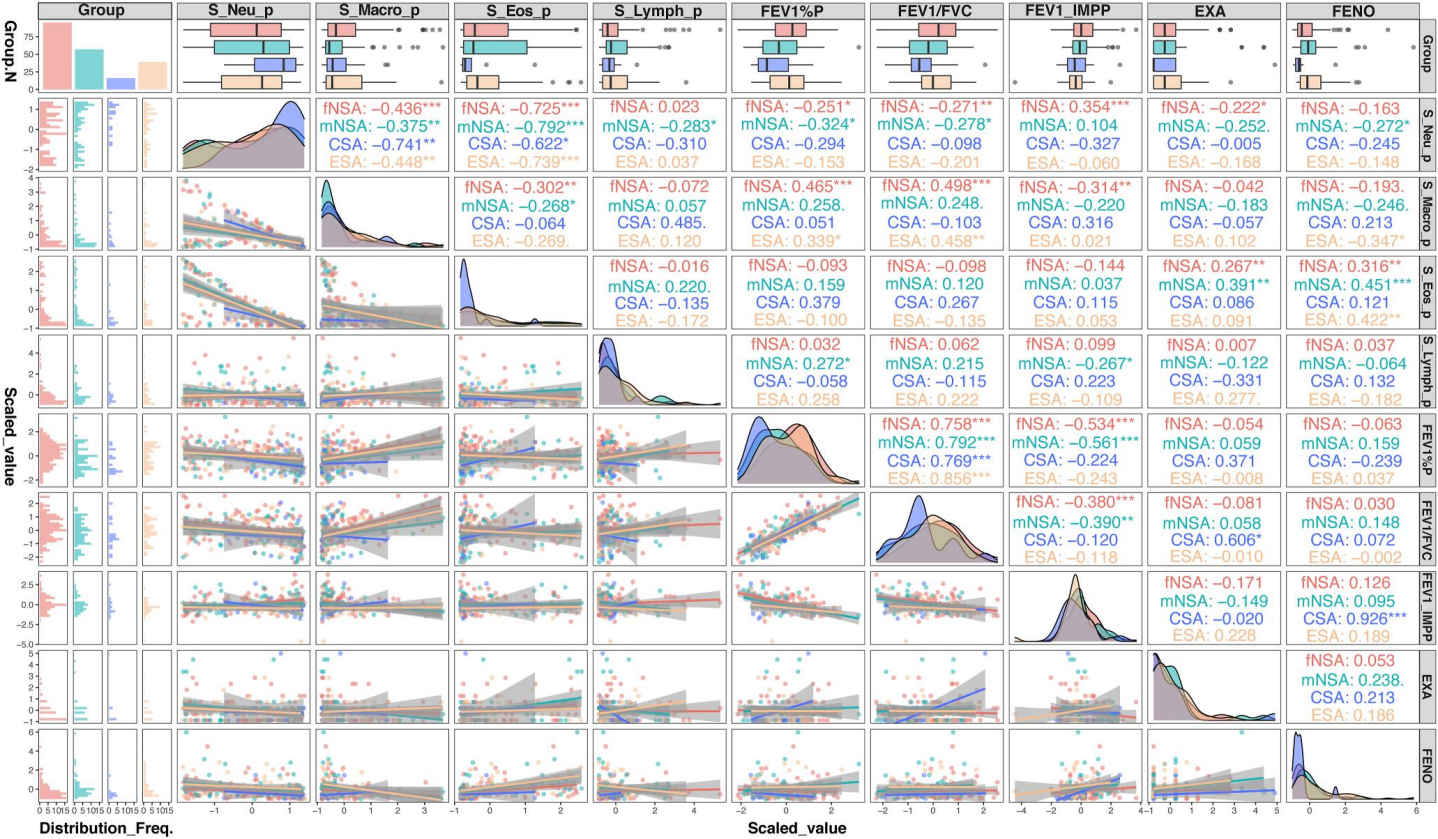
Correlation analyses for the three cohorts of severe asthma: non‐smoking severe asthma (NSA, *N* = 152), current‐smoking severe asthma (CSA, *N* = 16) and Ex‐smoking severe asthma (ESA, *N* = 39). The correlation analysis was performed for the whole group (“Corr”), followed by CSA (pink), ESA (green) and NSA (blue), using Spearman correlation analysis. The graphic relationship of these parameters are shown together with the actual correlations and their significance (*p < 0.05,**p < 0.01,***p < 0.001). The top row shows the median (25%–75% Interquartile range). EXA, Exacerbations in previous year; FENO, Fractional exhaled nitric oxide; FEV1%Pred, Pre‐bronchodilator FEV1%Predicted; FEV1/Forced Vital Capacity (FVC), Pre‐bronchodilator FEV1/FVC; FEV1IMPP, Post‐bronchodilator improvement in FEV1%Predicted; fNSA, female non‐smoking severe asthma; mNSA, male non‐smoking severe asthma; S_Eos_p, sputum eosinophil percentage; S_Lymph_p, sputum lymphocyte percentage; S_Macro_p, sputum macrophage percentage; S_Neu_p, sputum neutrophil percentage

At 1 year, in the NSA female, there was an improvement in ACQ5 and in total AQLQ reflected in the symptom, activity limitation and emotional domains, accompanied by an improvement in pre‐bronchodilator FEV_1_ (% predicted) from 67.8% to 72.23% (*p* = 0.066) (Figure 1C). In both male and female groups in NSA, there was a significant reduction in the bronchodilator response from 18.7% to 12.0% (*p* = 0.002) and from 21.50% to 12.15% (*p* < 0.001), respectively. There was no change in pre‐ and post‐bronchodilator FEV_1_ (% predicted), ACQ and AQLQ and FeNO and blood eosinophil counts in the CSA and ESA male participants (Figure 1D).

Non‐smoking severe asthma women had a lesser degree of airflow obstruction with lower levels of FeNO, despite similar blood eosinophil counts compared to male NSA. This is in contrast to the report of a higher disease burden of asthma in women compared to men.^5^ It may be because of the post‐menopause (as female NSA were aged 54 years) who were not obese and who have a lower age‐adjusted risk of asthma than premenopausal women.^6^, ^7^ There is also the consideration that asthma sometimes improves after the menopause.^8^ At 1 year, FEV_1_ in women improved, together with asthma control and quality of life measures, an indication of the continued benefits of the post‐menopausal period on asthma. Unfortunately, we did not collect any information on the menopause or the use of hormone replacement therapy in the women.

Currently‐smoking severe asthma patients subjects had lower eosinophilic inflammation, likely to be a suppressive effect of current smoking, with higher sputum neutrophilia.^9^ However, the degree of neutrophilia was related to airflow obstruction in NSA as previously reported.^10^ Interestingly, the level of eosinophilic inflammation was correlated with the number of exacerbations in the previous year, supporting its link with Type‐2 inflammation.^11^ In the C‐BIOPRED cohort, we have shown that sex differences and the effect of smoking are important influences on the treatable traits of severe asthma, with a likelihood of improvement in females at 1 year follow‐up and lower type‐2 inflammatory markers in currently‐smoking asthmatics. Importantly, our data indicate that in severe asthma, while neutrophilic inflammation may be linked to airflow obstruction, eosinophilia is associated with exacerbation rates.

## AUTHOR CONTRIBUTIONS

**Cong Dong**: Conceptualization (Equal), Formal analysis (Equal), Visualization (Equal), Writing – original draft (Equal). **Xiaojing Yang**: Data curation (Equal), Validation (Equal). **Wei Luo**: Methodology (Equal), Resources (Equal). **Ethan Fan**: Formal analysis (Equal), Investigation (Equal), Resources (Equal), Software (Equal), Visualization (Equal). **Nkouibert Pryseley Assam**: Data curation (Equal), Funding acquisition (Equal), Investigation (Equal), Resources (Equal). **Jian Kang**: Data curation (Equal), Investigation (Equal), Resources (Equal). **Yunhui Zhang**: Data curation (Equal), Investigation (Equal), Resources (Equal). **Mao Huang**: Data curation (Equal), Investigation (Equal), Resources (Equal). **Jinfu Xu**: Data curation (Equal), Investigation (Equal), Resources (Equal). **Kewu Huang**: Data curation (Equal), Investigation (Equal), Resources (Equal). **Qiang Li**: Data curation (Equal), Investigation (Equal), Resources (Equal). **Xiangyan Zhang**: Data curation (Equal), Investigation (Equal), Resources (Equal). **Jianping Zhao**: Data curation (Equal), Investigation (Equal), Resources (Equal). **Xiaoxia Liu**: Data curation (Equal), Investigation (Equal), Resources (Equal). **Shenghua Sun**: Data curation (Equal), Investigation (Equal), Resources (Equal). **Huaping Tang**: Data curation (Equal), Investigation (Equal), Resources (Equal). **Bei He**: Data curation (Equal), Investigation (Equal), Resources (Equal). **Shaoxi Cai**: Data curation (Equal), Investigation (Equal), Resources (Equal). **Ping Chen**: Data curation (Equal), Investigation (Equal), Resources (Equal). **Chunhua Wei**: Data curation (Equal), Investigation (Equal), Resources (Equal). **Guangfa Wang**: Data curation (Equal), Investigation (Equal), Resources (Equal). **Ping Chen**: Data curation (Equal), Investigation (Equal), Resources (Equal). **Lixin Xie**: Data curation (Equal), Investigation (Equal), Resources (Equal). **Jiangtao Lin**: Data curation (Equal), Investigation (Equal), Resources (Equal). **Yuling Tang**: Data curation (Equal), Investigation (Equal), Resources (Equal). **Zhihai Han**: Data curation (Equal), Investigation (Equal), Resources (Equal). **Xiuhua Fu**: Data curation (Equal), Investigation (Equal), Resources (Equal). **Changzheng Wang**: Data curation (Equal), Investigation (Equal), Resources (Equal). **Hua‐Hao Shen**: Data curation (Equal), Investigation (Equal), Resources (Equal). **Meiling Jin**: Data curation (Equal), Investigation (Equal), Resources (Equal). **Lei Zhu**: Data curation (Equal), Investigation (Equal), Resources (Equal). **Guochao Shi**: Data curation (Equal), Investigation (Equal), Resources (Equal). **Zhongmin Qiu**: Data curation (Equal), Investigation (Equal), Resources (Equal). **Zhongguang Wen**: Data curation (Equal), Investigation (Equal), Resources (Equal). **Wei Gu**: Data curation (Equal), Investigation (Equal), Resources (Equal). **Kian Fan Chung**: Investigation (Equal), Methodology (Equal), Project administration (Equal), Supervision (Equal), Validation (Equal), Writing – original draft (Equal), Writing – review & editing (Equal). **Qingling Zhang**: Investigation (Equal), Project administration (Equal), Writing – review & editing (Equal). **Nanshan Zhong**: Project administration (Equal), Supervision (Equal), Writing – review & editing (Equal).

## CONFLICT OF INTEREST

The authors declare no conflict of interest.

## CONSENT

All participants gave written and signed informed consent.

## FUNDING INFORMATION

Astra‐Zeneca China.

## Supporting information

Supporting Information S1
