# Supplementary material for: Influence of sex, cigarette smoking and airway inflammation on treatable traits in CBIOPRED severe asthma
Source: Clin Transl Allergy. 2022 Sep 15;12(9):e12189. doi: 10.1002/clt2.12189 (PMC9478422; doi:10.1002/clt2.12189)
Supplement: Supplementary file 1 — Supporting Information S1 [file CLT2-12-e12189-s001.docx]

| **Table S1 Demographic Characteristics and Biomarker Analysis** | | | | | | |
| --- | --- | --- | --- | --- | --- | --- |
|  | **Male** | | | **Female** | **P Value** | |
|  | **Current Smoker (N=41)** | **Ex-smoker (N=65)** | **Never Smoker (N=101)** | **Never Smoker (N=241)** | **P Value*** | **P Value**** |
| Gender |  |  |  |  |  |  |
| Male | 41 (100.0) | 65 (100.0) | 101 (100.0) | 0 (0.0) | 1.000 | <0.001 |
| Female | 0 (0.0) | 0 (0.0) | 0 (0.0) | 241 (100.0) |  |  |
| Age (years) | 59.70 (51.20,61.90) | 59.50 (53.10,64.30) | 53.70 (43.80,62.60) | 55.90 (49.10,61.30) | 0.015 | 0.319 |
| Age of diagnosis (years) | 47.40 (36.00,54.40) | 49.50 (38.30,58.50) | 41.60 (24.25,49.88) | 44.65 (31.40,52.60) | 0.012 | 0.167 |
| BMI (kg/m2) | 24.77 (23.05,27.82) | 24.98 (23.31,26.44) | 24.24 (22.76,26.03) | 23.83 (21.80,26.72) | 0.170 | 0.592 |
| BMI Normal (<28) | 31 (75.6) | 55 (84.6) | 88 (87.1) | 205 (85.1) | 0.246 | 0.736 |
| BMI Obesity (>=28) | 10 (24.4) | 10 (15.4) | 13 (12.9) | 36 (14.9) |  |  |
| Smoking (Pack-years) | 25.00 (15.00,40.00) | 21.25 (11.88,33.75) | NA | NA | 0.554 | NA |
| pre-BD FVC, L | 3.13 (2.54,3.67) | 3.04 (2.50,3.53) | 3.31 (2.65,3.65) | 2.27 (1.94,2.64) | 0.570 | <0.001 |
| pre-BD FVC % pred, L | 80.41±19.02 | 80.19±15.06 | 78.42±16.53 | 85.53±17.33 | 0.727 | <0.001 |
| pre-BD FEV1, L | 1.65 (1.32,2.22) | 1.62 (1.22,2.12) | 1.68 (1.25,2.36) | 1.46 (1.07,1.75) | 0.746 | <0.001 |
| pre-BD FEV1% pred | 59.93 (41.14,78.33) | 56.57 (43.50,74.84) | 56.52 (43.29,70.02) | 68.12 (51.99,82.61) | 0.710 | <0.001 |
| pre-BD FEV1/FVC, % | 58.27±13.35 | 55.56±12.14 | 55.69±11.35 | 62.05±12.21 | 0.516 | <0.001 |
| Exacerbations in prior year | 1.27±1.83 | 1.44±1.49 | 1.56±1.90 | 1.47±1.76 | 0.619 | 0.605 |
| Exacerbation in prior year,n(%) |  |  |  |  |  |  |
| Yes | 25 (61.0) | 44 (67.7) | 67 (66.3) | 152 (63.1) | 0.769 | 0.612 |
| No | 15 (36.6) | 20 (30.8) | 31 (30.7) | 82 (34.0) |  |  |
| Healthcare resource utilization,n(%) |  |  |  |  |  |  |
| Yes | 2 (4.9) | 12 (18.5) | 22 (21.8) | 42 (17.4) | 0.042 | 0.695 |
| No | 39 (95.1) | 53 (81.5) | 79 (78.2) | 199 (82.6) |  |  |
| Post-BD, n | 40 | 65 | 98 | 231 |  |  |
| Post-BD FEV1, L | 2.01 (1.60,2.63) | 1.94 (1.52,2.51) | 2.02 (1.53,2.72) | 1.67 (1.33,2.00) | 0.753 | <0.001 |
| Post-BD FEV1% pred | 68.25 (52.89,87.84) | 67.45 (52.97,84.59) | 67.01 (51.37,79.99) | 79.63 (66.32,94.03) | 0.689 | <0.001 |
| Post-BD FEV1(%) Improvement | 15.62 (8.64,23.94) | 15.77 (12.29,28.43) | 18.65 (12.22,25.65) | 20.50 (12.98,30.82) | 0.240 | 0.326 |
|  |  |  |  |  |  |  |
| Subjects with FeNO, n | 36 | 63 | 95 | 225 |  |  |
| FeNO(ppb) | 18.50 (13.00,33.50) | 37.00 (21.50,68.00) | 39.00 (24.50,66.50) | 28.00 (19.00,52.00) | <0.001 | 0.001 |
| >=30ppb | 11 (30.6) | 37 (58.7) | 63 (66.3) | 105 (46.7) |  |  |
| <30ppb | 25 (69.4) | 26 (41.3) | 32 (33.7) | 120 (53.3) | 0.001 | 0.001 |
| Subjects with blood results, n | 40 | 64 | 101 | 240 |  |  |
| Neutrophil count(10^9/L) | 3.90 (3.20,4.43) | 4.39 (3.42,5.46) | 3.73 (2.81,4.67) | 3.71 (2.99,4.60) | 0.042 | 0.925 |
| Eosinophil count(10^9/L) | 0.21 (0.10,0.33) | 0.30 (0.18,0.52) | 0.24 (0.12,0.49) | 0.23 (0.11,0.42) | 0.203 | 0.652 |
| Subjects with IgE and ECP results, n | 30 | 56 | 85 | 196 |  |  |
| ECP*(μg/L) | 7.61 (5.06,16.98) | 7.80 (4.30,13.85) | 7.05 (3.92,18.00) | 7.30 (4.14,13.33) | 0.710 | 0.811 |
| IgE Total (KU/L) | 189.50  (95.05,456.25) | 256.50 (100.00,539.75) | 193.00  (62.60,418.00) | 155.50  (57.50,392.00) | 0.337 | 0.637 |
| Atopy¹ |  |  |  |  |  |  |
| above normal | 21 (70.0) | 26 (46.4) | 39 (45.9) | 98 (50.0) | 0.060 | 0.603 |
| normal | 9 (30.0) | 30 (53.6) | 46 (54.1) | 98 (50.0) |  |  |
|  |  |  |  |  |  |  |
| Sputum cell count, n | 16 | 39 | 57 | 95 |  |  |
| Neutrophils (%) | 81.2 [57.3, 90.3] | 58.6 [28.6, 80.8] | 57.8 [21.8, 84.0] | 53.7 [18.8, 78.3] | 0.0788 | 0.7753 |
| Macrophages (%) | 11.0 [ 4.9, 22.0] | 6.3 [ 3.5, 32.1] | 6.3 [ 3.2, 16.8] | 11.9 [ 5.0, 26.8] | 0.7916 | 0.1074 |
| Eosinophils (%) | 4.3 [ 2.5, 9.3] | 17.0 [ 5.7, 32.8] | 15.2 [ 3.0, 59.9] | 12.8 [ 3.9, 41.0] | 0.0696 | 0.8809 |
| Lymphocytes (%) | 0.8 [ 0.3, 1.0] | 0.9 [ 0.2, 1.9] | 0.8 [ 0.5, 1.9] | 0.5 [ 0.2, 1.2] | 0.6329 | 0.0363 |
| BD: Bronchodilator, FEV1: Forced Expiratory Volume in one second, FVC: Forced Vital Capacity, N: Number of subjects in the cohort, n: Number of subjects included in the analysis, NA: Not applicable. SD: Standard deviation. FeNO: Fractional level of nitric oxide in exhaled breath, ECP: Eosinophil Cationic Protein. Note: Data shown as mean ± Standard deviation or median (interquartile range) for continuous variables or n (%) for categorical variables. The subject level data used for FVC, pre-BD FEV1, pre-BD FEV1% pred, pre-BD FEV1/FVC ratio, analysis follows the algorithm below: If baseline data is available then baseline data is used, or else if screening data is available, then screening data is used. p value*: Comparison is between three male groups.  p value**: Comparison is between male and female in never-smokers. Note: Kruskal-Wallis test is used for continuous data if it is not normally distributed, otherwise ANOVA is used for more than 2 category comparison, t test is used for pairwise comparison, Fisher exact test is used for discrete data.  ¹Atopy (above normal) means the subjects have at least one above normal (>0.35) results for allergens HX2, FX5, MX2, TX4, WX5 and for Phadiatop. | | | | | | |

| **Table S2 Maintenance Medication, Questionnaire scores and Comorbidities** | | | | | | |
| --- | --- | --- | --- | --- | --- | --- |
|  | **Male** | | | **Female** | **P Value** | |
|  | **Current smoker (N=41)** | **Ex-smoker (N=65)** | **Never smoker(N=101)** | **Never Smoker (N=241)** | **P value*** | **P value**** |
| Subject with questionnaire, n | 41 | 64 | 101 | 241 |  |  |
| ACQ |  |  |  |  |  |  |
| ACQ5 | 2.00 (1.40,2.40) | 1.80 (1.15,2.60) | 1.60 (1.00,2.40) | 1.60 (1.00,2.40) | 0.360 | 0.685 |
| ACQ7 | 2.00 (1.57,2.71) | 2.00 (1.54,2.71) | 1.86 (1.29,2.57) | 1.86 (1.14,2.43) | 0.475 | 0.587 |
| AQLQ |  |  |  |  |  |  |
| Total | 4.81 (4.28,5.31) | 4.48 (3.56,5.17) | 4.53 (3.94,5.47) | 4.47 (3.62,5.22) | 0.114 | 0.103 |
| Symptoms | 4.83 (4.17,5.42) | 4.62 (3.73,5.60) | 4.67 (4.00,5.58) | 4.67 (3.92,5.50) | 0.433 | 0.383 |
| Activity limitation | 4.82 (4.36,5.55) | 4.27 (3.43,5.39) | 4.55 (3.91,5.36) | 4.36 (3.55,5.09) | 0.041 | 0.100 |
| Emotional | 4.80 (3.80,6.00) | 4.40 (3.40,5.40) | 4.60 (3.60,5.60) | 4.20 (3.40,5.20) | 0.292 | 0.048 |
| Environmental | 4.75 (4.00,5.50) | 4.50 (2.75,5.31) | 4.50 (3.50,5.50) | 4.00 (3.00,5.00) | 0.130 | 0.054 |
| ESS | 8.00 (5.00,11.00) | 7.00 (4.00,9.00) | 7.00 (4.00,9.00) | 6.00 (4.00,9.00) | 0.411 | 0.199 |
| MARS | 21.00 (20.00,24.00) | 23.00 (20.00,24.00) | 22.00 (20.00,24.00) | 22.00 (20.00,24.00) | 0.648 | 0.360 |
| Any comorbidities | 27 (65.9) | 44 (67.7) | 63 (62.4) | 161 (66.8) | 0.405 | 0.359 |
| Allergic rhinitis | 16 (39.0) | 27 (41.5) | 45 (44.6) | 119 (49.4) | 0.822 | 0.478 |
| Eczema | 10 (24.4) | 17 (26.2) | 16 (15.8) | 35 (14.5) | 0.207 | 0.742 |
| GERD | 9 (22.0) | 9 (13.8) | 12 (11.9) | 36 (14.9) | 0.294 | 0.500 |
| Hay fever | 9 (22.0) | 10 (15.4) | 7 (6.9) | 28 (11.6) | 0.028 | 0.242 |
| Nasal polyps | 12 (29.3) | 15 (23.1) | 22 (21.8) | 54 (22.4) | 0.670 | 1.000 |
| Rhinitis, not specified | 5 (12.2) | 11 (16.9) | 9 (8.9) | 28 (11.6) | 0.305 | 0.568 |
| Sinusitis | 3 (7.3) | 7 (10.8) | 10 (9.9) | 17 (7.1) | 0.908 | 0.386 |
| Any surgical history | 5 (12.2) | 9 (13.8) | 15 (14.9) | 34 (14.1) |  |  |
| Nasal polypectomy | 5 (12.2) | 7 (10.8) | 12 (11.9) | 27 (11.2) | 1.000 | 0.853 |
| Sinus operation | 2 (4.9) | 2 (3.1) | 7 (6.9) | 12 (5.0) | 0.612 | 0.450 |
|  |  |  |  |  |  |  |
| Any asthma medication | 41 (100) | 65 (100) | 101 (100) | 241 (100) | NA | NA |
| Antibiotic | 1 (2.44) | 1 (1.54) | 6 (5.94) | 13 (5.39) | 0.410 | 0.801 |
| Antihistamine | 0 | 2 (3.08) | 3 (2.97) | 13 (5.39) | 0.710 | 0.412 |
| Chinese traditional medicine | 3 (7.32) | 12 (18.46) | 11 (10.89) | 34 (14.11) | 0.132 | 0.486 |
| ICS/LABA | 41 (100) | 64 (98.46) | 101 (100) | 241 (100) | NA |  |
| Immunostimulants[a] | 0 | 1 (1.54) | 0 | 2 (0.83) | 0.512 | 1.000 |
| Injected glucocorticoids[b] | 1 (2.44) | 1 (1.54) | 4 (3.96) | 1 (0.41) | 0.860 | 0.028 |
| LAMA | 10 (24.39) | 19 (29.23) | 20 (19.8) | 34 (14.11) | 0.353 | 0.196 |
| Mucolytics | 3 (7.32) | 3 (4.62) | 1 (0.99) | 14 (5.81) | 0.102 | 0.077 |
| Nasal steriod | 0 | 0 | 0 | 1 (0.41) | 1.000 | 0.504 |
| OCS[c] | 4 (9.76) | 10 (15.38) | 13 (12.87) | 21 (8.71) | 0.709 | 0.241 |
| SABA | 4 (9.76) | 9 (13.85) | 11 (10.89) | 36 (14.94) | 0.844 | 0.391 |
| SABA/SAMA | 1 (2.44) | 1 (1.54) | 0 | 4 (1.66) | 0.261 | 0.324 |
| SAMA | 1 (2.44) | 2 (3.08) | 0 | 4 (1.66) | 0.189 | 0.324 |
| Singulair/LTRA | 19 (46.34) | 31 (47.69) | 50 (49.5) | 118 (48.96) | 0.949 | 1.000 |
| Xanthines | 3 (7.32) | 16 (24.62) | 18 (17.82) | 40 (16.6) | 0.070 | 0.755 |
| Data are presented as median (quartile range) for not normally distributed variables of questionnaire scores. For cormobidities and medication, data shown as number (percent of total).  ACQ5 or ACQ7=Asthma Control Questionnaire with 5 or 7 questions; AQLQ=Asthma Quality of Life Questionnaire; ESS=Epworth sleepiness scale; MARS=Medication adherence response scale. ICS, inhaled corticosteroids; LABA, long-acting beta-agonist; LAMA, long-acting muscarinic antagonist; LTRA, leukotriene receptor antagonist; ND, Not done; OCS, oral corticosteroid; SABA, short-acting beta-agonist; SAMA, Short-acting muscarinic receptor antagonist. N=Number of subjects in the cohort. n=Number of subjects included in the analysis. p value*: Comparison is among the three male groups. p value**: Male vs. Female in never-smokers.Kruskal-Wallis test is used.   [a] Immunostimulants include Bacterial Lysates, Spleen Aminopeptide and Oral Lyophilized Powder. [b] Subjects who take medication with intravenous for glucocorticoids have been classified as injection glucocorticoids. [c] Subjects who take medication with oral glucocorticoids have been classified as OCS. | | | | | | |
